# Cytogenetic Consequences of Food Industry Workers Occupationally Exposed to Cooking Oil Fumes (COFs)

**DOI:** 10.31557/APJCP.2021.22.11.3591

**Published:** 2021-11

**Authors:** Manikantan Pappuswamy, Arun Meyyazhagan, Balamuralikrishnan Balasubramanian, Haripriya Kuchi Bhotla, Karthika Pushparaj, Murugesh Easwaran, Vijaya Anand Arumugam, Thirunavukkarasu Periyaswamy, Aditi Chaudhary, Nanditha Rajesh, Rajkumar Sundaram, Karthick Dhandapani

**Affiliations:** 1 *Department of Life Sciences, CHRIST (Deemed to be University), Bangalore, Karnataka, India. *; 2 *Department of Food Science and Biotechnology, College of Life Science, Sejong University, Seoul 05006, Republic of Korea. *; 3 *School of Engineering, Shanghai Jiao Tong University, Shanghai, 200241, China. *; 4 *Department of Zoology, School of Bioscience, Avinashilingam Institute for Home Science and Higher Education for Women, Coimbatore- 641 043, Tamil Nadu, India. *; 5 *Computational Biology Laboratory, Department of Bioinformatics, Bharathiar University, Coimbatore-641046, Tamil Nadu, India. *; 6 *Department of Human Genetics and Molecular Biology, Bharathiar University, Coimbatore-641046, Tamil Nadu, India. *; 7 *Nehru Arts & Science College (Affiliated to Bharathiar University), Coimbatore, Tamilnadu, India. *; 8 *Bharathiar University, Coimbatore, Tamilnadu, India.*

**Keywords:** Cooking Oil Fumes, Cytogenetic Changes, DNA, Comet, Tobacco user

## Abstract

**Background:**

Cooking oil fumes (COFs) with smoking habits is a substantial risk that aggravates genetic modifications. The current study was to estimate the biological markers of genetic toxicity counting Micronucleus changes (MN), Chromosome Aberrations (CA) and DNA modifications among COFs exposures and control subjects inherent from South India.

**Materials and Methods:**

Present analysis comprised 212 COFs with tobacco users and equivalent number of control subjects.

**Results:**

High frequency of CA (Chromatid type: and chromosome type) were identified in group II experimental subjects also high amount of MN and DNA damage frequency were significantly (p < 0.05) in both subjects (experimental smokers and non-smokers). Present analysis was observed absence of consciousness among the COFs exposures about the destructive level of health effects of tobacco habits in working environment.

**Conclusion:**

COFs exposed workers with tobacco induce the significant alteration in chromosomal level. Furthermore, a high level of rate of genetic diseases (spontaneous abortion) were identified in the experimental subjects. This finding will be helpful for preventive measures of COFs exposed workers and supportive for further molecular analysis.

## Introduction

Respiratory related diseases are foremost causing of ill effects and deaths to human population (Jemal et al., 2011). Moreover, recent studies focused that occupational hazard increased the genetic damage with smoking habitats. (Smyth., 1996). Mostly these hazarded chemicals directly affect the respiratory system. However, the incidence and death rate of lung cancer is now declining in most of the individual exposed to COFs (Yin et al., 2009). COFs are common, everyday household inhalant that induces by carcinogens (Chiang et al., 1999). 

COFs are increased significant genetic changes in human health as it indorses physical stress that shows a vital role to inducing the physiological changes (Ewart Toland et al., 2004). Effects of COFs can cause acute mucosal irritation of the eyes, nose and throat, very recently studies have noted carcinogenic fumes in household environment increase the risk of cancer in prostate (Ewart Toland et al., 2004), esophageal (Jin et al., 2008), breast (Shin et al., 2005), gastric (Jin et al., 2007) and lung organs system (Park et al., 2006). 

Recent analysis also showed that COFs induce a genetic change like inhibit the protein and cellular level apoptotic changes to cause the lung cancer (Kim et al., 2015; Chiang et al., 1999). It’s evident that COFs causes racial and local differences in genetic traits play an important role in the pathophysiology of lungs (Zhu et al., 2001; Srivastava et al., 2010). Polycyclic aromatic hydrocarbons are known genotoxic agents react with DNA to produce genotoxic effects finally cause the cancers (Ng et al., 2014). Numerous studies evaluated the genotoxic (Rodu et al., 2004), carcinogenicity and deleterious health effects of COFs (Xue et al., 2016) but, no attempt has been made till date to evaluate the harmful consequences of these agents. CA analysis has been significant biomarker to detect the changes in DNA level (Herceg and Hainaut., 2007). 

Oral cancer (Jiang et al, 2019) was induced by tobacco related habitual and it’s been evaluated and classified as genotoxic to human beings (Young et al, 2010), reports are not available for COFs with tobacco induct the genetic damage. In addition, comet assay is highly valuable techniques to estimate genetic toxicity from people exposed to toxic chemicals and to identify the various kinds of DNA damage including single strand breaks (Collins, 2004). Numerous investigations have noted, exposure to COFs surge risk of lung cancer (Shen et al., 2014) because it contains numerous mutagens and carcinogens (Tung et al., 2001) and increased oxidative tension in exposed subjects to COFs (Svecova et al., 2009) through the valuation factor such as oxidative DNA damage. Genetic variations in gene level discrepancy in DNA repair capacity may lead to carcinogen (Mei Wu et al, 2008) these features are increased cancer susceptibility in individual person. Moreover, molecular epidemiology is an innovative approach to early detection of the health risks due to occupational environmental exposure. It allows for the direct determination of occupational effects caused by the contact with toxic substances by genotoxic agents (Bonassi et al., 2005). A high frequency of MN is a biomarker of genotoxic assessment also, can be detect the loss and breaks of chromosome.

The cytogenetic parameters are performed to analyze the toxic hazards to selected growing cells. (Bonetta et al., 2019: Ceretti et al., 2014). In the last three decades, MN analyses have been widely used in molecular genetic analysis (Hayashi, 2016). They are considered as biomarkers of early detection that are formed in the cells due to alterations of the chromosomal structure and oxidative stress characteristics to numerous other factors, among which occupational exposure (Kirsch-Volders, 2011). MN analysis can also be detected i various tissues depending on the causative factors to be evaluated. In past few years, exfoliated epithelial cells from buccal mucosa have been increasingly used to detect MN frequency related to atmospheric exposure (Arul et al., 2018). 

Buccal mucosa (BM) is a very sensitive tissue, directly exposed to airborne pollutants and also easily isolate from exposed subjects (Thomas et al., 2009). Moreover, the identification of genotoxic end-points in dividing cells for the assessment of cytogenetic damage from blood or epithelial cells (Fenech, 2007). Indeed, the MN frequency could be modulated by other demographical factors such as genetics, lifestyle, and individual health status. Some studies found out to date (Zona et al., 2019) took into consideration only the epidemiological data and found an increase in mortality for some cancers. The focal aim of the present study was estimating the level of genotoxic effects of COFs exposed workers from various types of food industry through CA, MN and comet assays.

## Materials and Methods

The present analysis was carried out totally 212 individuals working in various food industries, hotel, bakes and other food product preparation units in and around Southern India. All the subjects were grouped based on the types and usage of tobacco stuffs as TS (Cigarettes and Beedi) and SLT (areca nut and betel quid etc.) users and equal number of age and sex matched healthy controls (212) also selected for present analysis. 

Approval for ethical has been approved by committee. Hand-written knowledgeable agreement (Annexure file) was obtained from all the subject individuals prior to collecting circulated peripheral blood and exfoliated buccal cells. Duration of exposure, tobacco usage, other health complaints (Abortion, High blood pressure, Diabetes and Heart issues) and mode of working history was noted using health questionnaire of the study populations, 94 were males’ subjects and 104 were female subjects. Further, the subjects were categorized into two groups such as group I and group II based on the age and duration of exposure to COFs I (<10 years of exposures) with an age range from 15 to 35 years and group II (>10 years of exposures) above 35 years of age.

*Collection of blood sample*

Blood was separated from control and experimental subjects and transferred to heparin coated container for CA, MN and DNA analysis. Same manner had been followed for buccal cell from buccal cavity for MN analysis.

*Micronucleus analysis in peripheral blood lymphocytes*

Micronucleus assay was performed by Fenech and Morley (1986). In short, RPMI-1640 medium added in to 20% Fetal Bovine serum (FBS), 0.2 mL of Phytohemagglutinin (PHA) and 2mM L-Glutamine supplements. Mixed thoroughly and added 0.5 mL of peripheral venous blood. Finally incubated at 37°C for 72-74 hours. Added cytochalasin B after the incubation at 44 hrs for blocking cytokinesis reactions, and final concentration was maintained of 6 μg/mL. Treated with 5 mL of 0.075 M KCL (hypotonic solution) for 15 minutes after the cells were harvested at 72 hrs of incubation. Added Methanol:Glacial Acetic Acid in 3:1 ratio as a fixative solution. Fixative treatment was repeated for 2 or 3 times after storage at 4 °C and ultimately stained in Giemsa stain. 

*Micronucleus analysis in exfoliated epithelial buccal cells*

Collected the buccal cells by scraping the cheek with fresh spatula after rinsing the mouth with distilled water. Added and stored in 0.9% NaCl (Saline) solution. Cell were separated by centrifugation of 800 rpm for 5 minutes, added fixative (methanol: glacial acetic acid in 3:1 ratio) and cells were dropped onto cleaned slides. After the incubation for drying the slides were stained with Feulgen plus solution. Identified and screened the solution according to Saro et al., (1987).

*Study of chromosome aberrations*

Chromosome assay was performed by Hoyos et al., (1996). In short, RPMI-1640 medium added in to 20% Fetal Bovine serum (FBS), 0.2 mL of Phytohemagglutinin (PHA), 100 U/mL of penicillin and 100 μg/mL streptomycin and 2mM L-Glutamine supplements. Mixed thoroughly and added 0.5 mL of peripheral venous blood. Finally incubated at 37°C for 71-72 hrs. Added 0.01 mg/mL Colcemid after the incubation at 44 hrs for arrest the cells at mitotic stage, and final concentration was maintained of 6 μg/mL. Culture was treated with 5 mL of 0.075 M KCL (hypotonic solution) for 15 minutes after the cells were harvested at 72 hrs of incubation. Added Methanol:Glacial Acetic Acid in 3:1 ratio as a fixative solution. Fixative treatment was repeated for 2 o 3 times after storage at 4°C and ultimately stained in giemsa stain. Metaphase chromosome were analyzed and viewed under light microscope with 100X magnification.

*Analysis of DNA damage by single cell gel electrophoresis or Comet assay*

DNA damage analyzed according to Tice et al., (1992). Fresh blood cells were lysed by adding of lysis solution with lysis solution containing 140 μL of proteinase K at room temperature for 2 hrs. prepared slides were placed on horizontal gel electrophoresis. Unwind the DNA by 10-15 minutes in running buffer solution at 25 V and 300 mA. Slides were removed and maintain the pH by adding with 0.4 M Tris-HCl, pH 7.5. EtBr solution slide and the cover glass was placed over the gel. DNA damage was measured by visualization of cells into grouping of comets present in the tail. Comet tail length was measured in random wise manner. The fluorescence microscope (Labomed) was prepared with a BP546/12-nm excitation 590-nm barrier filter for analysing the DNA damage (Tail length (TL) and tail moment (TM). 

*Statistical analysis*

Statistical examination (one-way ANOVA) was carried out using the statistical software (SPSS Version 16) and p < 0.05 was considered as a significance level.

*Human blood samples collection statement*

Present study confirmed that all experiments were performed in accordance with relevant guidelines and regulations. The work was followed and carried out in accordance with the guidelines laid down in the 1964 Declaration of Helsinki.

## Results

Totally 212 (93 males, 119 females) personals were selected for this present study. Both control and experimental subjects were classified into two groups (group I and II) based on the year of exposure period. Among the individual subjects, based on the health questionnaire duration of exposure and smokeless tobacco usage was high in female subjects than male subjects. Furthermore, heart related disease, diabetic, blood pressure and spontaneous abortion also found out during the sample collections (Table 1).

*Micronucleus frequency in blood and buccal epithelial cells*

Blood MN analysis was showed significantly (p < 0.05) increased in group I experimental subjects (1.64 ± 0.87) when compared to their respective controls matched with age and sex (0.69 ± 0.54). On the other hand, group II experimental subjects also showed that significantly higher level of MN frequency (2.37 ± 0.67) than control subjects (1.09 ± 0.56). Moreover, combined results showed that higher frequency of MN in smoking and smokeless tobacco user (2.50 ± 0.93) than personal having individual habits like smoking (2.11 ± 0.72) and smokeless tobacco (2.21 ± 0.9 87) subjects (Table 2).

**Figure 1:**
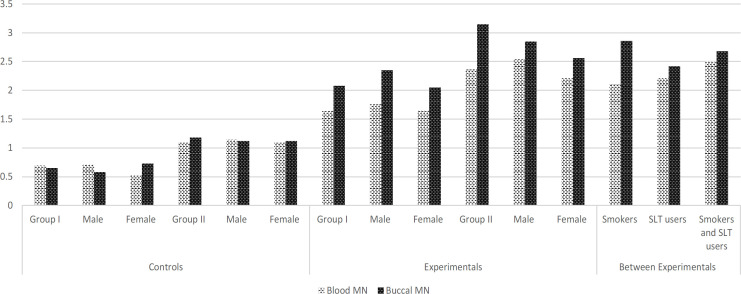
Represent the Frequencies of Micronuclei in Buccal and Blood Cells of COFs Exposures and Controls. Blood MN Level was Elevated when Compared to the Controls and Other Subjects

Exfoliated buccal epithelial cell MN analysis showed that significantly (p < 0.05) higher in both group I (2.09±1.31) and group II (3.15±1.27) experimental subjects than control subjects. Moreover, combined results showed that higher frequency of MN in smoking and smokeless tobacco user than personal having individual habits like smoking and smokeless tobacco subjects (Table 2). Addition to that, present analysis found out that higher frequency of MN analysis found in group II males (both blood and buccal epithelial cells) than females. The frequency was showed statistically significant (p < 0.05) results (Figure 1).

*Chromosome aberration analysis*

**Figure 2:**
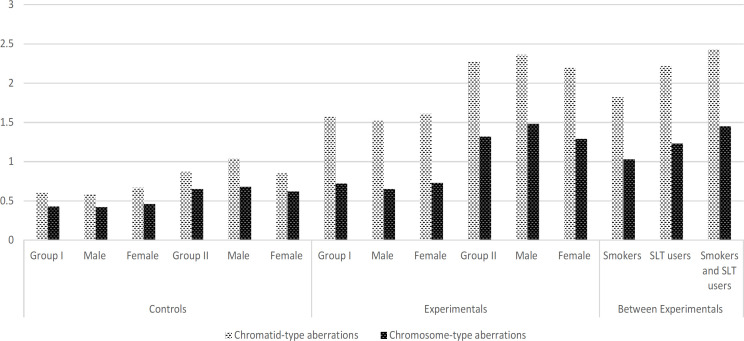
Shows the Chromosome Alterations in Controls and Experimental Subjects

A significantly increased number of chromatid gaps and breaks (Minor abnormalities) were observed in group I (1.57 ± 0.84) and group II (2.28 ± 1.21) experimental subjects when compared with group I (0.60 ± 0.77) and group II (0.87 ± 0.60) control subjects respectively. Moreover, significantly higher number of Chromosome aberrations found in in group I (0.72 ± 0.64) and group II (1.32 ± 1.15) experimental subjects than in group I (0.43 ± 0.40) and group II (0.65 ± 0.60) controls subjects (Figure 2). On the other hand, among the experimental subjects, higher level of chromosomal aberrations (Chromatid type 2.43 ± 1.28 and Chromosome type 1.45 ± 0.84) was noted in both users (smoke and smokeless tobacco) than individuals’ user.

*DNA damage by Single cell gel electrophoresis or Comet assay*

**Figure 3:**
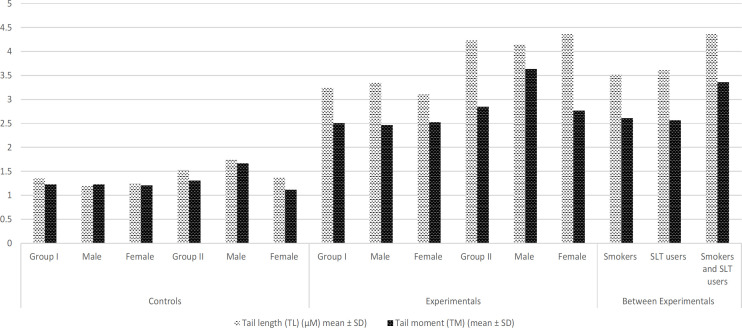
Demonstration of Comets Scored in Blood Cells of COFs Exposures and Controls

Significantly higher number of DNA Tail Length (TL) and Tail Moment (TM) was detected in experimental subject group I (TL: 3.25 ± 1.25 and TM: 2.51 ± 1.27) and group II (TL: 4.24 ± 1.43 and TM: 2.85 ± 1.32) than control subjects. Additionally, significantly increased number of DNA damage (both TL and TM) in both habit like smoking and smokeless tobacco users (TL: 4.37 ± 1.13 and TM: 3.36 ± 1.49) than respective control subjects (Table 4 and Figure 3).

**Table 1:** Demographic Details of Age and Year of Exposure and Risk Factors in Tobacco Users

Particulars	No. of sample and %	Year of exposure Mean ± SD	Age Mean ± SD	BP (%) (Blood Pressure) RR :120/80	Diabetes (%)	Spontaneous Abortion	Cardiac Complaint
Total Experimentals	212	10.63 ± 4.86	42.69 ± 14.67	33 (15.56)	23 (10.84)	7 (3.30)	5 (2.35)
Male	93 (43.86)	11.63 ± 5.83	41.44 ± 15.12	11 (11.82)	10 (10.75)		3 (3.22)
Female	119 (55.66)	10.21 ± 4.19	43.39 ± 14.40	12 (10.08)	13 (10.92)	7 (5.88)	2 (1.68)
Controls							
Group I	84 (39.62)		31.19 ±7.46		1 (1.19)		
Male	37 (44.04)		28.63 ± 5.68				
Female	47 (55.95)		32.23 ± 8.18		1 (2.12)		
Group II	128(60.37)		52.22 ± 7.47	4 (3.12)	4 (3.12)		
Male	56 (43.75)		54.25 ± 9.32	2 (3.57)	2 (3.57)		
Female	72 (56.25)		52.70 ± 8.01	2 (2.77)	2 (2.77)		
Experimental							
Group I	84 (39.62)	6.15 ± 0.75	29.20 ± 5.49	3 (3.57)	5 (5.95)	09 (10.71)	
Male	37 (44.04)	5.48 ± 1.20	26.79 ± 5.63	1 (2.70)	2 (5.40)		
Female	47 (55.95)	6.46 ± 1.69	31.54 ± 4.87	2 (4.25)	3 (6.38)	09 (19.14)	
Group II	128(60.37)	15.56 ± 3.56	52.45 ± 12.38	22 (17.18)*	17 (8.01)		4 (03.12)
Male	56 (43.75)	16.03 ± 4.61	50.42 ± 12.38	12 (21.48)	8 (14.28)		3(5.35)
Female	72 (56.25)	13.60 ± 3.10	53.43 ± 12.51	10 (13.88)	09 (12.50)		1 (1.38)
Between Experimental	212						
Smokers	53 (25.00)	10.74 ± 5.89	34.67 ± 13.81	2(3.77)	2(3.77)		
SL tobacco users	104 (49.05)	11.23 ± 4.29	40.85 ± 13.09	06 (5.76)	6 (5.76)	3 (2.88)	
Smokers and SLT users	55 (25.94)	13.86 ± 4.49	58.55 ± 09.54	15 (27.27)*	05(09.09)		1(1.81)

SD, standard deviation; FS, Female subjects; RR, Reference range; *, Significantly higher when compared to the other groups.

**Table 2:** Frequencies of Micronuclei Scored in Buccal and Blood Cells of Tobacco Users and Controls

Particulars	No. of subjects studied	Mean frequency of micronuclei ± standard deviation of MN scored in blood cells/1000 (mean ± SD)	Mean frequency of micronuclei ± standard deviation of MN scored in buccal cells/2000 (mean ± SD)	Blood p < 0.005	Buccal p < 0.005
Controls					
Group I	84	0.69 ± 0.54	0.65 ± 0.58		
Male	37	0.71 ± 0.57	0.58 ± 0.58	0.003	
Female	47	0.53 ± 0.52	0.73± 0.62		0.021
Group II	128	1.09 ± 0.56	1.18 ± 0.71	0.001	0.34
Male	56	1.14 ± 0.58	1.12 ± 0.65		
Female	72	1.09 ± 0.56	1.12 ± 0.74		0.002
Experimental					
Group I	84	1.64 ± 0.87	2.08 ± 1.31*	0.001	
Male	37	1.76 ± 0.65	2.35 ± 1.38		0.001
Female	47	1.65 ± 0.96	2.05 ± 1.42		0.703
Group II	128	2.37 ± 0.67^#^	3.15 ± 1.27*		0.001#
Male	56	2.54 ± 0.71**	2.85 ± 1.39	0.002	0.001
Female	72	2.21 ± 0.80	2.56 ± 1.35		
Between Experimentals	212				
Smokers	53	2.11 ± 0.72	2.86 ± 1.46		0.002
SLT users	104	2.21 ± 0.87	2.42 ± 1.23		
Smokers and SLT users	55	2.50 ± 0.82^a^	2.68 ± 1.53	0.002	0.001

*,^ #^, Significantly elevated when compared to controls subjects as estimated by ANOVA; **, Significantly elevated compared to controls and group I and female experimental subjects. A Significantly elevated compared to controls and smokers experimental subject as estimated by ANOVA followed by Bonferroni’s correction for multiple comparisons.

**Table 3:** Frequencies of Chromosome Aberrations in Smokeless Tobacco Users and Controls

Subjects	Number of samples	Chromosome aberrations		Chromatid- type aberrations p < 0.005	Chromosome type aberration p < 0.005
		Chromatid- type aberrations (mean ± SD)	Chromosome-type aberration (mean ± SD)		
Controls					
Group I	84	0.60 ± 0.77	0.43 ± 0.40		
Male	37	0.58 ± 0.68	0.42 ± 0.41	0.324	1
Female	47	0.67 ± 0.78	0.46± 0.43		
Group II	128	0.87 ± 0.60	0.65 ± 0.60		
Male	56	1.04 ± 0.65	0.68 ± 0.76		0.02
Female	72	0.86 ± 0.61	0.62 ± 0.57	0.042	0.801
Experimental					
Group I	84	1.57 ± 0.84	0.72 ± 0.64		
Male	37	1.52 ± 0.85	0.65 ± 0.72	0.001	
Female	47	1.61 ± 0.80	0.73 ± 0.64		0.325
Group II	128	2.28 ± 1.21#	1.32 ± 1.15		0.002
Male	56	2.36 ± 1.09	1.48 ± 0.86		
Female	72	2.20 ± 1.08	1.29 ± 1.28	0.002	0.324
Between Experimentals	212				
Smokers	53	1.82 ± 1.03	1.03 ± 0.83		
SLT users	104	2.22 ± 1.42	1.23 ± 1.31	0.001	
Smokers and SLT users	55	2.43 ± 1.28*^a^	1.45 ± 0.84*^a^		0.001

*, Significantly elevated when compared to the controls and smokers’ subjects as estimated by ANOVA; ^#^, Significantly elevated compared to the group I subjects; ^a^, Significantly elevated when compared to controls and smokers experimental subject as estimated by ANOVA followed by Bonferroni’s correction for multiple comparisons.

**Table 4:** Frequencies of Comets Scored in Blood Cells of Tobacco Users and Controls

Subjects	Number of samples	Tail length (TL) (μM) mean ± SD)	Tail moment (TM) (mean ± SD)
Controls			
Group I	84	1.36 ± 1.15	1.23 ± 0.65
Male	37	1.20 ± 0.82	1.23 ± 0.72
Female	47	1.25 ± 1.23	1.21 ± 0.63
Group II	128	1.53 ± 1.42	1.31 ± 1.08
Male	56	1.74 ± 1.01#	1.67 ± 1.26^a^
Female	72	1.37 ± 0.78	1.12 ± 1.93
Experimental			
Group I	84	3.25 ± 1.25	2.51 ± 1.27
Male	37	3.35 ± 1.24	2.47 ± 1.50
Female	47	3.12 ± 1.24	2.53 ± 1.17
Group II	128	4.24 ± 1.43*	2.85 ± 1.32
Male	56	4.15 ± 1.52	3.64 ± 0.34
Female	72	4.37 ± 1.46^#^	2.77 ± 0.52
Between Experimentals	212		
Smokers	53	3.52 ± 1.46	2.61 ± 1.42
SLT users	104	3.62 ± 1.54	2.57 ± 1.14
Smokers and SLT users	55	4.37 ± 1.13**	3.36 ± 1.49

*p < 0.001 level significantly; elevated compared to controls groups. #Significantly elevated when compared to controls and group I; experimental male and female subject. **, Significantly elevated when compared to controls and smokers and SLT; experimental subject as estimated by ANOVA followed by Bonferroni’s correction for multiple comparisons.

## Discussion

Experiments on genotoxicity found that the genetic damage was slightly higher in COFs exposed subjects than control subjects. personals using tobacco product in both the way induce the genetic damage in significant level higher. The way of packing in different mode has been increased the genetics as mutagenic (Chang et al., 1987). In our study pointed out that combined effect of COFs with tobacco exposure has high number of genetic modifications noted in both blood and buccal epithelial cells. Recently high level of genetic damage including mutation level in DNA strand has been noted (DeMerini, 2004).

According to the statement given by Gupta et al., (2018) direct correlation between chewing tobacco with oral cancer. In same way present study also detected significant level of MN was increase in COFs workers than controls. This level has been increased due to tobacco products contains high level of genotoxic agents (Hecht, 2012). Same manner Bonaasi et al., (2003) also reported significant level of increased in smokers than non-smokers. Moreover, higher level MN frequencies identified in exfoliated buccal epithelial cells (Kausar et al., 2009). This studies more or less similar with recent epidemiological studies (Haveri et al., 2010; Cavallo et al., 2007; Kamboj and Mahajan, 2007). Our, studies have pointed out that the buccal MN cell also significantly higher in COFs exposed worker than controls subjects. 

Report stated by Orta and Gunebakan (2012) age factor also increased the higher number of MN in subjects exposed to mutagen. Studies identified that tobacco users may loss some chromosomal regions like 8p, 9p, 9q, 17q and 20q (Lin et al., 2002) but it might be higher in tobacco users and it also associated with cancer (Rossner et al., 2005; Bonassi et al., 2006). Present study also found out the higher number of CA and MN in aged subject than control and younger group of subjects. According to Fenech et al., (1998) age factor also caused the significant level increased in MN and CA. Hence, age factor and duration of exposures are paly an important role in present study (Cigerci et al., 2015). According to Tang et al., (2010) smoking is directly correlated with smoking personal in COFs industry with high level of genetic damage. Similarly smoking may cause and induce and progress to cancer (Hecht, 2008;) in industry with exposure of COFs (Mannix et al., 1996). Present study also showed that high frequency of genetic changes noted in experimental subjects than controls. 

Comet assay have been established genotoxic analysis to determine the toxic compound in working environment (Liman et al., 2015). Recent reports stated that (Jayakumar and Saikala, 2008) extensive use of working environment which cause the genetic damage in common population. Current analysis showed that DNA mismatching occurred in experimental subjects than controls. Hence the present study is needed to assess these exposures on higher genetic level. This confirmation that COFs may have a mutual effect with smoking habits family history for convinced similar living environments conditions. However, no association between these two nonetheless, infuriate COFs several co-efficient factors that modulate the higher genotoxicity level in smokers than non-smokers (Chen and Lee, 1996). Harmful particulates matter show in COFs industry which cause the genotoxic damage in workers (Chiang et al., 1999;Pan et al., 2008). In this present study also experimentally proved that COFs induce the mutagenicity and carcinogenicity.

In accumulation to having carcinogenic properties, acquaintance to chemicals from COFs industry may cause innate immunity impairment and antioxidant imbalances (Olloquegui and Silva, 2016) even lung cancer risk (Ko et al., 2000) in addition with recent case-control studies (Chen et al., 2020; Saha et al., 2005; Wu et al., 2010). Respiratory symptoms also caused by cigarette smoking with occupational exposure personal working in COFs industry. Indeed, in smokers had been exposed to a lifetime chronic bronchitis than others (Wu et al., 2004) with our detailed analysis (Table 2; Table 3) with smoking habits. 

Moreover, numerous studies have certain limitation (Srivastava et al., 2010). On the other hand, our results may prove that genotoxicity and carcinogenicity with exposed COFs (Srivastava et al., 2010, Halliwell and Gutteridge, 2015) also it may cause finally cellular death (Hecht, 2008). Carcinogens which are present in smoking products are known to activate proinflammatory responses and release of cytokines, production, and ultimately damage to DNA (Ohshima and Bartch, 1994), directly effect to DNA damage (Szeto et al., 2009) and other biomolecules when inhaled (Fenech and Bonassi, 2011). Our findings were consistent with other previous investigations regarding the relationship between diet and genotoxic DNA damage, numerous studies took into considerate effect of individual nutrients on MN formation (Villarini et al., 2018) other studies considered the combination of nutrients and the dietary models (Peng et al., 2015), who highlighted an increase of MN in the buccal mucosa cells due to genotoxic agents.

XRCC1 and COMT gene polymorphisms and COFs exposure are central risk factors in the progression of lung cancer (Yu et al., 2006). Current study analyzed those possible effects of en-vironmental risk factors such as smoking with COFs exposure and chromosomal in DNA level relations among COFs exposure. The outcomes of a recent study have also suggested that exposure to COFs was similar to elevated level increased in lung cancer among workers exposed to COFs (Jin et al., 2015). Besides, the risk factor of COFs exposure continues to be related with lung diseases (Yin et al., 2014). Finally, above the mentioned factors increased the genetic variant of COFs risk factors with various confounding factors such as ST SLT.

In conclusion, present study showed that substantial level of genetic damage caused by the COFs factors with smoking habitats. Demographic factors such as age and exposed duration further induce higher level of toxic effects in genetic level to experimental subjects and it would be benefit to impressionist the other kind of segmental population. Further molecular analysis needed to be confined this relationship to explore the factors which are causing genetic damage with COFs workers. 

## Author Contribution Statement

None.
